# MycPermCheck: the *Mycobacterium tuberculosis *permeability prediction tool for small molecules

**DOI:** 10.1186/1758-2946-5-S1-P21

**Published:** 2013-03-22

**Authors:** Benjamin Merget, David Zilian, Tobias Müller, Christoph A Sotriffer

**Affiliations:** 1 Institute of Pharmacy and Food Chemistry, University of Würzburg, Am Hubland, D-97074, Würzburg, Germany; 2 Department of Bioinformatics, Biocenter, University of Würzburg, Am Hubland, D-97074, Würzburg, Germany

## 

With over 8 million new cases in 2010, particularly documented in developing countries, tuberculosis (TB) is still a highly present pandemic and often terminal [1]. This is also due to the emergence of antibiotic-resistant strains (MDR-TB and XDR-TB) of the primary causative agent *Mycobacterium tuberculosis *(MTB). Efforts to develop new effective drugs against MTB are restrained by the unique and largely impermeable mycobacterial cell wall [2]. Accordingly, tools for estimating the ability of chemical compounds to pass this barrier would significantly support TB drug discovery.

Using the CDD TB database of antimycobacterial substances [3], 3,815 active and, thus, permeable compounds could be retrieved. A data mining approach was conducted to gather the similarities of these substances in terms of physico-chemical descriptors and to delimit them from a generic dataset of drug-like molecules. Principal component analyses were performed and a logistic regression model was generated on the basis of the differences of these datasets in the first principal component. This regression model was thoroughly evaluated using highly active MTB inhibitors absent in the training dataset, as well as inhibitors of the validated MTB drug target InhA. Based on these validation results, its performance is expected to be of high practical value for virtual screening purposes. The regression model was implemented into the online tool MycPermCheck to predict the permeability probability of small organic compounds against the MTB cell wall. Given the current lack of precise molecular criteria determining mycobacterial permeability, MycPermCheck represents an unprecedented knowledge-driven prediction tool intended to support antimycobacterial drug discovery. It can be used intuitively as an additional selection criterion for potential new inhibitors against MTB. The MycPermCheck online tool is freely accessible under the URL: http://www.mycpermcheck.aksotriffer.pharmazie.uni-wuerzburg.de.
